# Identification of Candidate Genes that Affect the Contents of 17 Amino Acids in the Rice Grain Using a Genome-Wide Haplotype Association Study

**DOI:** 10.1186/s12284-023-00658-9

**Published:** 2023-09-15

**Authors:** Xiaoqian Wang, Lihong Xie, Jiachuang Fang, Yunlong Pang, Jianlong Xu, Zhikang Li

**Affiliations:** 1 State Key Laboratory of Crop Biology, Shandong Key Laboratory of Crop Biology, College of Agronomy, Shandong Agricultural University, 271018 Tai’an, China; 2 State Key Laboratory of Rice Biology and Chinese National Center for Rice Improvement, National Rice Research Institute, 310006 Hangzhou, China; 3 Institute of Crop Sciences, National Key Facility for Crop Gene Resources and Genetic Improvement, Chinese Academy of Agricultural Sciences, 100081 Beijing, China; 4 Shenzhen Branch, Guangdong Laboratory for Lingnan Modern Agriculture, Genome Analysis Laboratory of the Ministry of Agriculture, Agricultural Genomics Institute at Shenzhen, Chinese Academy of Agricultural Sciences, 518120 Shenzhen, Guangdong China

**Keywords:** GWAS, Genome-wide haplotype association study, Amino acid content, Candidate genes

## Abstract

**Background:**

The amino acid content (AAC) of the rice grain is one of the most important determinants of nutritional quality in rice. Understanding the genetic basis of grain AAC and mining favorable alleles of target genes for AAC are important for developing new cultivars with improved nutritional quality.

**Results:**

Using a diverse panel of 164 accessions genotyped by 32 M SNPs derived from 3 K Rice Genome Project, we extracted 1,123,603 high quality SNPs in 44,248 genes and used them to construct haplotypes. We measured the contents of the 17 amino acids that included seven essential amino acids and 10 dispensable amino acids. Through a genome-wide haplotype association study, 261 gene-trait associations containing 174 genes for the 17 components of AAC were detected, and 34 of these genes were associated with at least two components. Furthermore, the associated SNPs in genes were also identified by a traditional genome-wide association study to identify the key natural variations in the specific genes.

**Conclusions:**

The genome-wide haplotype association study allowed us to detected candidate genes directly and to identify key natural genetic variation as well. In the present study, twelve genes have been cloned, and 34 genes were associated with at least two components, suggesting that the genome-wide haplotype association study approach used in the current study is an efficient way to identify candidate genes for target traits. The identified candidate genes, favorable haplotypes, and key natural variations affecting AAC provide valuable resources for further functional characterization and genetic improvement of rice nutritional quality.

**Supplementary Information:**

The online version contains supplementary material available at 10.1186/s12284-023-00658-9.

## Background

Rice (*Oryza sativa* L.) is the predominant stable food for approximately one-third of the world’s population. Rice feeds more than half of the world’s population and accounts for over 25% of the daily caloric intake for these consumers (Kusano et al. 2015). Rice is an important source of nutrition and energy, especially for poor people in developing countries who are solely dependent on rice as a staple food crop (Birla et al. 2017; Yang et al. 2016). Therefore, it is crucial to enhance the nutritional quality of rice to improve consumers’ nutrition and health (Huang et al. 2023).

The amino acid content (AAC) of the rice grain is one of the most important determinants of rice nutritional quality (Shi et al. 2023), and AAC plays a key role in the maintenance of healthy and sustainable human diets and food systems (Adhikari et al. 2022). On the basis of nutritional needs from the diet to meet optimal requirements for humans, amino acids (AAs) have traditionally been classified as essential amino acids (EAAs) and dispensable amino acids (DAAs). EAAs cannot be synthesized by the human body, or are inadequately synthesized de novo, and they must be obtained from our diet to meet optimal requirements, whereas DAAs can be synthesized de novo in adequate amounts by the human body to meet optimal requirements (Wu 2009). There are nine EAAs that include valine, leucine, isoleucine, phenylalanine, tryptophan, threonine, lysine, methionine, and histidine, all of which are essential for optimal growth and development of humans or monogastric animals (Galili et al. 2016). DAAs are required for the synthesis of EAAs and for normal cellular physiology and metabolism, and DAAs are also important factors that determine nutritional quality (Wu 2009). Therefore, it is important to characterize the genetic basis of AAC and to mine favorable alleles for AAC to improve the nutritional quality of rice.

AAC is quantitatively inherited, and by mining quantitative trait loci (QTLs), some genes or main effect QTLs (M-QTLs) for AAC have been detected on all chromosomes except chromosomes 5 and 12. Shi et al. (2023) performed a genome-wide association study (GWAS) and detected a gene, *OsAUX5*, that encodes a transmembrane amino acid transporter that functions in the uptake of multiple AAs, and controls multiple EAAs (valine, leucine, and phenylalanine) on chromosome 11. Using a population of 134 recombinant inbred lines (RILs) from a cross between ‘Dasan-byeo’ (a Tongil-type *xian* line) and TR22183 (a temperate *geng* line), Yoo (2017) identified six M-QTLs on chromosome 3 and 26 epistatic QTLs on nine chromosomes for six components of AAC. A RIL population comprising 188 lines derived from a cross between ‘Zhenshan 97B’ and ‘Delong 208’ was used to detect the QTLs for protein content and 17 components of AAC. A total of 48 and 64 M-QTLs were identified in 2004 and 2005, respectively, and three major QTL clusters were mapped on chromosomes 1, 7, and 9 (Zhong et al. 2011). Lu et al. (2009) constructed a population of 241 RILs from a cross between ‘Zhenshan 97’ and ‘Minghui 63’ to detect QTLs for 17 components of AAC. A total of 12 M-QTLs were identified and one QTL cluster on chromosome 1 was associated with the contents of eight AAs. Using a population of 190 RILs from a cross between ‘Zhenshan 97’ and ‘Nanyangzhan’, Wang et al. (2008) identified 18 QTLs for 19 components of AAC, and one QTL cluster consisting of up to 19 individual QTLs was identified at the bottom of chromosome 1. The AA metabolism in plants has been reported previously which would be helpful for gene mining for AAC and improvement of rice nutritional quality by genetic engineering (Binder 2010; Mahender et al. 2016). For example, lysine is regarded as the most important EAA, and the low levels of lysine limit the nutritional value of cereal grains (Ufaz and Galili 2018). The enzymes aspartate kinase (AK) and dihydrodipicolinate synthase (DHPS) involved in AA synthesis, and lysine-ketoglutarate reductase/saccharopine dehydrogenase (OsLKR/SDH), which participates in AA catabolism, are key enzymes in the regulation of Lys metabolism (Galili et al. 2005). Long et al. (2013) increased the free Lys levels in seeds ~ 60-fold by enhancing Lys biosynthesis via the expression of AK and DHPS, or down-regulating its catabolism by inhibiting rice OsLKR/SDH. Taiji (2010) decreased the levels of the lysine-degrading enzyme OsLKR/SDH by knocking down expression of the seed storage protein genes *RISBZ1* and *RPBF*, resulting in a significant increase in the content of free AAs and the lysine content in rice grains. Therefore, it is important to explore new genes/QTLs that regulate AAC in rice.

Although an increasing number of QTLs have been reported, only a few genes for AAC have been cloned. One primary reason is that cloning genes that affect quantitative traits is extremely time consuming using classical map-based QTL cloning. With the rapid development of next-generation DNA sequencing technology, GWAS, which uses high-density molecular markers and more diverse genetic resources, has proven to be a powerful approach to mine genes for quantitative traits in rice at an unprecedented rate (Shi et al. 2023; Zhao et al. 2018).

In the present study, we used genome-wide haplotype association (GWHA) approach to identify genes controlling AAC, which combined a genome-wide gene-based association study with haplotype analysis. We measured the contents of 17 AAs in the panel of 164 diverse rice accessions, and the GWHA study was conducted using the 32 M SNPs generated from the 3,000 Rice Genomes Project (3 K RGP) (Wang et al. 2018). The objectives of our study were to (1) evaluate the variation in AAC among the 164 rice accessions and (2) dissect the genetic architecture and identify candidate genes for AAC. Our study will provide valuable germplasms, useful candidate genes, and high-throughput SNP markers for AAC improvement in the breeding of high quality rice varieties.

## Materials and Methods

### Plant Materials and Field Trials

In the germplasm panel, 164 accessions with similar heading dates were selected from the 3 K RGP. These accessions originated from 42 countries or regions and comprised seven rice types including *Xian* (*indica*) (113 accessions), intermediate type (5), *Geng* (*japonica*) (13), temperate *Geng* (*japonica*) (16), tropical *Geng* (*japonica*) (12), *aus*/*boro* (2) and *basmati*/*sadri* (3) (Supplementary Table S1).

All of the rice accessions were grown at Sanya (18.3°N, 109.3°E) in Hainan province from Dec 2014 to Apr 2015, and we used a randomized complete block design with two replicates. Each accession was plated in a two-row plot with 10 plants per row at a spacing of 20 cm × 25 cm. The local farmers’ standard management practices were used in the field management. At maturity (about 40 days after flowering), eight representative plants in each plot were bulk harvested and air-dried for three months in the drying house.

### Phenotyping

The preparation of milled rice followed the methods described by Wang et al. (2017). The milled rice was then further ground into flour using a cyclone sample mill (model CT410, Foss, Denmark) and samples were stored at -20℃ prior to analysis. The contents of 17 AAs were determined with an AA autoanalyzer (model L-8800, Hitachi) using a method described in detail by Wang et al. (2008). In brief, approximately 100 mg of each sample was hydrolyzed with 50 ml of 6 mol/L hydrochloric acid (HCl) for 22 h at 110 °C. Then 1 ml of hydrolysate was transferred to a centrifugal tube and purified in a rotary evaporator to remove the HCl and water. The residue was completely dissolved in 1 ml of 0.02 M HCl and about 0.8 ml of supernatant was used to analyze the AAC by an AA autoanalyzer. A standard sample was used to calculate the amount of each component of AAC (g/kg). The assay for each accession was conducted with two replicates, and the average values were used for data analysis.

### SNPs for GWAS and Haplotype Analysis

The raw data for the 32 M SNPs were downloaded from the 3 K RGP in the Rice SNP-Seek Database (http://snp-seek.irri.org/). The SNPs within annotated genes were extracted based on gene functional annotations of the reference *Geng* (*japonica*) ‘Nipponbare’ genome IRGSP-1.0 from the Rice Genome Annotation Project (http://rice.plantbiology.msu.edu/). After removing SNPs with minor allele frequency (MAF) < 0.05 and missing data rate > 20% in the association panel, there were 1,123,603 SNPs remaining that were used to construct the gene haplotypes to conduct GWAS.

### Phenotypic and Population Structure Analyses

Phenotypic correlations were computed using the “chart.Correlation” function implemented in the R package PerformanceAnalytics (Peterson et al. 2014). The population structure and kinship were calculated using 8,367 evenly distributed SNPs extracted from the 1,123,603 SNPs with an average marker spacing of ~ 50 kb. The population structure was estimated by a model-based Bayesian clustering analysis method implemented in STRUCTURE software version 2.3.4 (Pritchard et al. 2000). We used 10,000 burnin iterations followed by 10,000 MCMC (Markov Chain Monte Carlo) iterations for each run, and nine independent simulations were run using K-values from 1 to 9. The Centered_IBS method implemented in TASSEL 5.2.23 was utilized to calculate the kinship (Bradbury et al. 2007).

### GWHA Study

To detect the candidate genes governing the components of AAC, the associations between gene haplotypes and AAC were analyzed using the “*lm*” function with the principal components to correct cryptic relatedness. Significant genes associated with the investigated traits were claimed when the test statistics reached *p* < 1.0 × 10^− 4^. The Manhattan plots and quantile-quantile plots were generated using the R package ‘‘qqMan’’. Traditional GWAS was also conducted to identify marker-trait associations using the 1,123,603 SNPs within annotated genes with the GAPIT (version 3.0) R package, and the SNPs with the lowest p-value in each significant gene were considered as the key natural variations of the gene.

## Results

### Phenotypic Variation and Trait Correlations

**Fig. 1:**
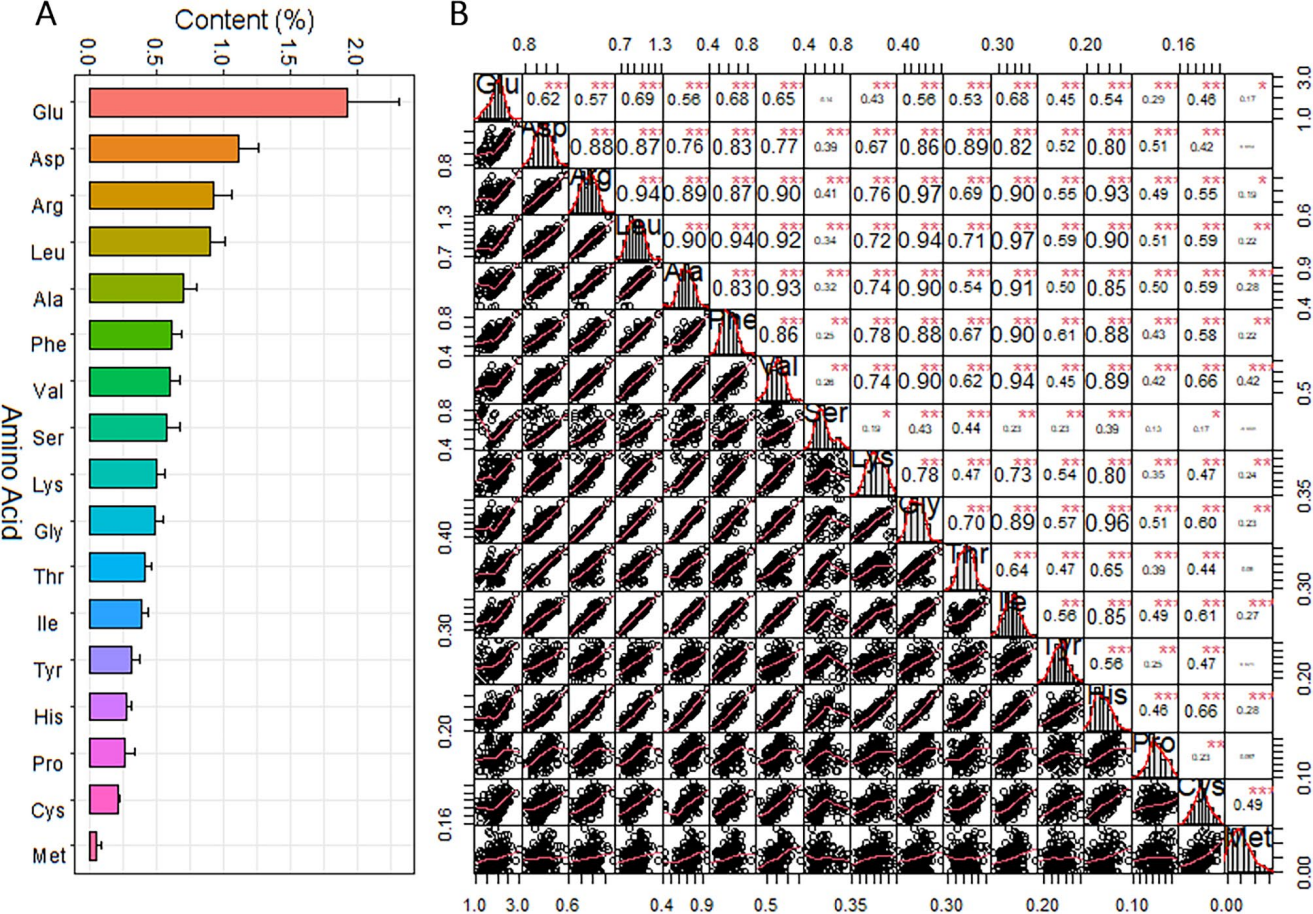
The amino acid contents (**A**) and correlations (**B**) among the 17 amino acids studied in the association panel

The contents of the 17 AAs varied widely in the association panel. The content of Glu was the highest, averaging 1.93%, followed by Asp, Arg, and Leu with average values of 1.12%, 0.93% and 0.90%, respectively. The average content of Met was the lowest at 0.05% (Fig. 1A). The AAC in the *Xian* and *Geng* subpopulations showed no significant difference in all components except for Cys (Supplementary Fig S1). The *Xian* subpopulation showed higher levels of variation in Ala, Arg, Asp, Glu, Lys, Met, Pro, Ser, and Thr, but less variation in Cys, Gly, Phe, Tyr, and Val than in the *Geng* subpopulation.

Most of the components of AAC appeared to be normally distributed showing largely consecutive variations, and most of the components showed significant positive correlations with each other, with the correlation coefficients ranging from 0.17 to 0.97. However, the correlations between Met and Asp, Ser, Thr, Tyr, Pro, and between Ser and Pro, Glu, were not significant (Fig. 1B). The top five rice accessions with the highest contents of each AA were selected, and 29 accessions were identified. Among them, three accessions, IRIS_313.10067, IRIS_313.8412, and CX237, had higher contents of 12 to 13AAs. Moreover, the total AAC of IRIS_313.8412 and CX237 were the highest at 14.12% and 14.04%, respectively. Six accessions (IRIS_313.11731, IRIS_313.7832, IRIS_313.7778, B051, IRIS_313.11796, and IRIS_313.9560) had high contents of 3–6 AAs with the total AA content ranging from 12.32% to 12.64%. The other 20 accessions had high contents of 1–2 AAs with the total AAC ranging from 9.65% to 12.08% (Table 1).

**Table 1:** The top five rice accessions with the highest contents of each of the 17 amino acids

ID	Asp	Thr	Ser	Glu	Gly	Ala	Cys	Val	Met	Ile	Leu	Tyr	Phe	Lys	His	Arg	Pro	Total
IRIS_313.8412	**1.56**	**0.56**	0.77	**3.02**	**0.66**	**0.95**	**0.28**	**0.80**	0.08	0.43	**1.29**	0.35	**0.86**	**0.62**	**0.39**	**1.27**	0.23	14.12
CX237	**1.54**	**0.53**	0.73	**2.81**	**0.66**	**0.96**	0.24	**0.83**	0.04	**0.54**	**1.26**	**0.43**	**0.82**	**0.64**	**0.38**	**1.34**	0.29	14.04
IRIS_313.9560	1.38	**0.51**	0.69	**2.63**	0.57	0.83	0.24	0.72	0.09	**0.49**	**1.15**	**0.44**	**0.74**	0.53	0.31	1.12	0.20	12.64
IRIS_313.11796	1.31	0.47	0.64	2.47	**0.59**	**0.88**	0.24	0.69	**0.13**	0.44	1.08	0.39	0.73	0.60	**0.36**	1.14	0.33	12.49
IRIS_313.7778	1.34	0.49	0.66	2.49	0.57	0.84	0.22	0.68	0.00	**0.47**	**1.10**	**0.43**	**0.76**	**0.61**	0.34	1.07	**0.42**	12.49
IRIS_313.7832	1.36	0.50	0.67	**2.56**	0.57	0.83	0.22	0.68	0.00	0.46	**1.11**	0.36	0.72	0.55	0.33	1.13	**0.43**	12.48
B051	**1.40**	**0.52**	0.67	2.46	**0.59**	0.79	**0.25**	0.69	0.06	0.46	1.07	0.40	0.73	0.60	**0.35**	**1.16**	0.21	12.41
IRIS_313.11731	1.25	0.47	0.63	2.49	0.57	**0.88**	0.23	**0.73**	0.06	**0.47**	1.09	0.33	**0.74**	0.58	0.32	1.08	**0.40**	12.32
IRIS_313.10067	**1.46**	**0.54**	0.67	1.74	**0.62**	**0.88**	**0.25**	**0.76**	0.09	**0.48**	**1.10**	0.36	**0.74**	**0.64**	**0.36**	**1.24**	0.35	12.28
IRIS_313.11297	1.25	0.46	0.63	2.40	0.56	0.81	**0.26**	**0.73**	0.11	0.47	1.06	0.35	0.67	0.56	0.33	1.10	0.33	12.08
IRIS_313.10333	**1.40**	0.50	0.65	2.38	0.56	0.78	0.22	0.65	0.04	0.43	1.04	0.38	0.69	0.55	0.33	1.05	**0.40**	12.05
IRIS_313.11802	1.22	0.45	0.62	2.43	0.54	0.85	0.22	0.72	0.08	**0.48**	1.09	0.34	0.73	0.55	0.31	1.05	0.37	12.05
CX225	1.34	0.50	0.63	**2.50**	0.56	0.82	0.22	0.70	0.04	0.44	1.05	0.32	0.69	0.53	0.31	1.06	0.29	12.00
IRIS_313.11307	1.19	0.47	0.60	2.29	0.54	0.78	**0.27**	0.66	**0.13**	0.43	0.97	0.34	0.66	0.57	0.32	1.02	0.26	11.50
IRIS_313.11120	1.15	0.44	0.58	2.13	0.53	0.77	0.23	0.64	0.10	0.41	0.95	**0.47**	0.60	0.57	0.32	0.99	**0.41**	11.29
IRIS_313.11261	1.14	0.44	0.59	2.34	0.51	0.76	**0.26**	0.68	0.10	0.42	0.98	0.30	0.66	0.50	0.30	0.98	0.29	11.25
IRIS_313.10967	1.26	0.47	0.67	1.53	0.58	0.82	0.23	0.69	0.09	0.43	1.01	0.24	0.64	0.60	**0.35**	**1.15**	0.28	11.04
IRIS_313.10224	1.26	0.47	0.67	1.63	0.53	0.80	0.24	0.66	0.04	0.45	1.04	0.34	0.68	0.51	0.30	1.00	**0.41**	11.03
IRIS_313.9922	1.31	**0.51**	0.67	1.62	0.55	0.76	0.23	0.64	**0.14**	0.41	0.98	0.26	0.67	0.52	0.32	1.03	0.39	11.01
IRIS_313.8571	1.26	0.48	0.67	1.58	0.52	0.75	0.22	0.65	**0.14**	0.41	0.97	0.25	0.66	0.50	0.30	1.00	0.39	10.75
B043	1.19	0.43	0.59	2.10	0.50	0.66	0.20	0.57	0.04	0.39	0.94	0.38	**0.75**	0.58	0.31	0.97	0.13	10.73
IRIS_313.9503	1.26	0.46	**0.88**	1.46	0.53	0.75	0.20	0.62	0.02	0.40	0.94	0.32	0.65	0.56	0.30	1.01	0.37	10.73
IRIS_313.11599	1.14	0.44	**0.87**	1.43	0.54	0.78	0.20	0.66	0.06	0.40	0.91	0.36	0.62	0.59	0.31	1.01	0.22	10.54
IRIS_313.10430	1.17	0.40	**0.89**	1.38	0.54	0.74	0.19	0.60	0.00	0.40	0.96	**0.45**	0.66	0.52	0.30	1.09	0.23	10.52
IRIS_313.11854	1.06	0.42	0.54	2.01	0.49	0.71	0.23	0.62	0.05	0.40	0.87	**0.47**	0.61	0.52	0.28	0.92	0.22	10.42
IRIS_313.10151	1.16	0.41	**0.86**	1.42	0.51	0.72	0.19	0.61	0.04	0.38	0.92	0.28	0.61	0.49	0.29	1.00	0.24	10.13
IRIS_313.8641	1.12	0.43	**0.84**	1.42	0.48	0.70	0.21	0.58	0.06	0.38	0.90	0.28	0.59	0.46	0.27	0.92	0.28	9.92
IRIS_313.11824	0.98	0.37	0.48	1.84	0.47	0.69	0.22	0.59	**0.15**	0.36	0.83	0.30	0.57	0.49	0.26	0.85	0.29	9.74
IRIS_313.9771	1.08	0.39	0.51	1.82	0.43	0.57	0.18	0.51	0.03	0.34	0.79	0.34	0.66	**0.64**	0.26	0.83	0.27	9.65

Note: The values in bold indicate the top accessions with the highest amino acid contents

### Basic SNP Marker Statistics

**Fig. 2:**
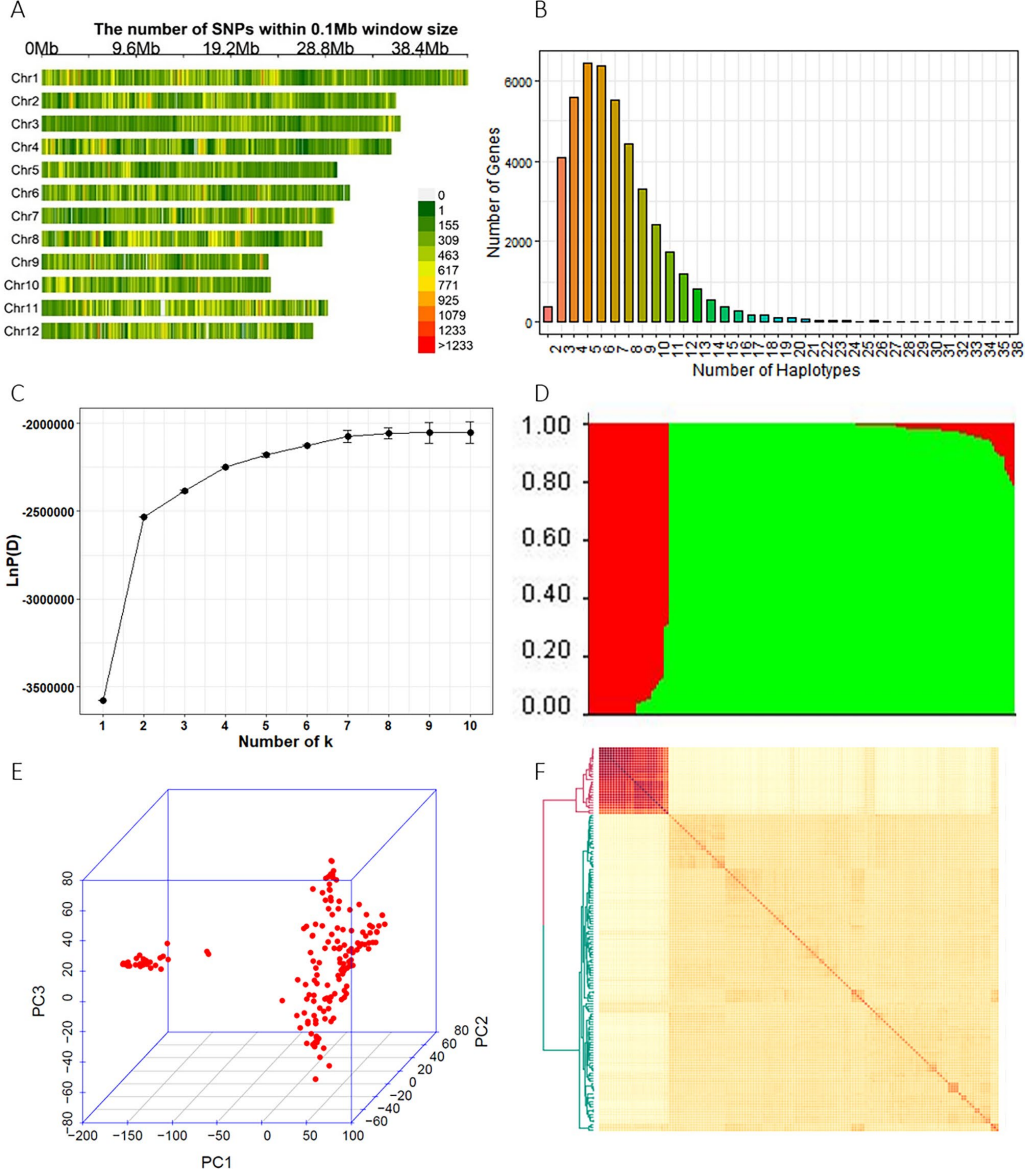
Genotype distribution and the population structure of the accessions in the rice association panel. (**A**) The distribution of SNPs with minor allele frequency > 0.05 and missing data rate ≤ 20% on the 12 rice chromosomes. (**B**) The number of haplotypes for the 44,248 genes covered by the SNPs. (**C**) Screen plot from the STRUCTURE analysis showing the selection of Q for the association study. (**D**) Bayesian clustering of the 164 accessions performed using STRUCTURE. (**E**) Population structure revealed by 3D principal component analysis. (**F**) Population structure revealed by kinship analyses

A total of 1,123,603 high quality SNPs identified in the whole population were used in the data analysis (Supplementary Table S2, Fig. 2A). The number of markers per chromosome ranged from 67,934 on chromosome 9 to 137,277 on chromosome 1. The size of the chromosomes varied from 22.9 Mb for chromosome 9 to 43.2 Mb for chromosome 1, and the whole genome size was 372.2 Mb. The genome-wide average marker spacing was 333.5 bp, varying from 281.3 bp for chromosome 11 to 380.7 bp for chromosome 5. These SNPs were located in 44,248 annotated genes across the whole genome, and the number of SNPs in these genes ranged from 1 to 591. These SNPs were used to construct haplotypes of the genes, and the number of haplotypes for these genes varied from 2 to 38. Among them, 35,749 genes had 3 to 8 haplotypes accounting for 73.3% of the total genes (Fig. 2A, B).

### Population Structure

From the screen plot generated by STRUCTURE, we observed that the ascent changed gradually when k > 2 which indicated that the 164 accessions could be divided into two distinct subpopulations (Fig. 2C, D). In addition, the kinship analysis and clustering analysis also showed that the current panel consisted of two subpopulations (Pop I and Pop II) (Fig. 2E, F). Pop I consisted of 40 accessions and most of them were temperate *Geng* (16), *Geng* (12), and tropical *Geng* (12). Pop II comprised 124 accessions and most them were *Xian* (114). In this panel, 56% (92/164) of the accessions did not show any admixture and 30% (50/164) showed less than 5% admixture, which indicated a distinct population structure in this rice panel (Fig. 2D).

### SNPs and Genes Associated with AAC Detected by GWAS

**Fig. 3:**
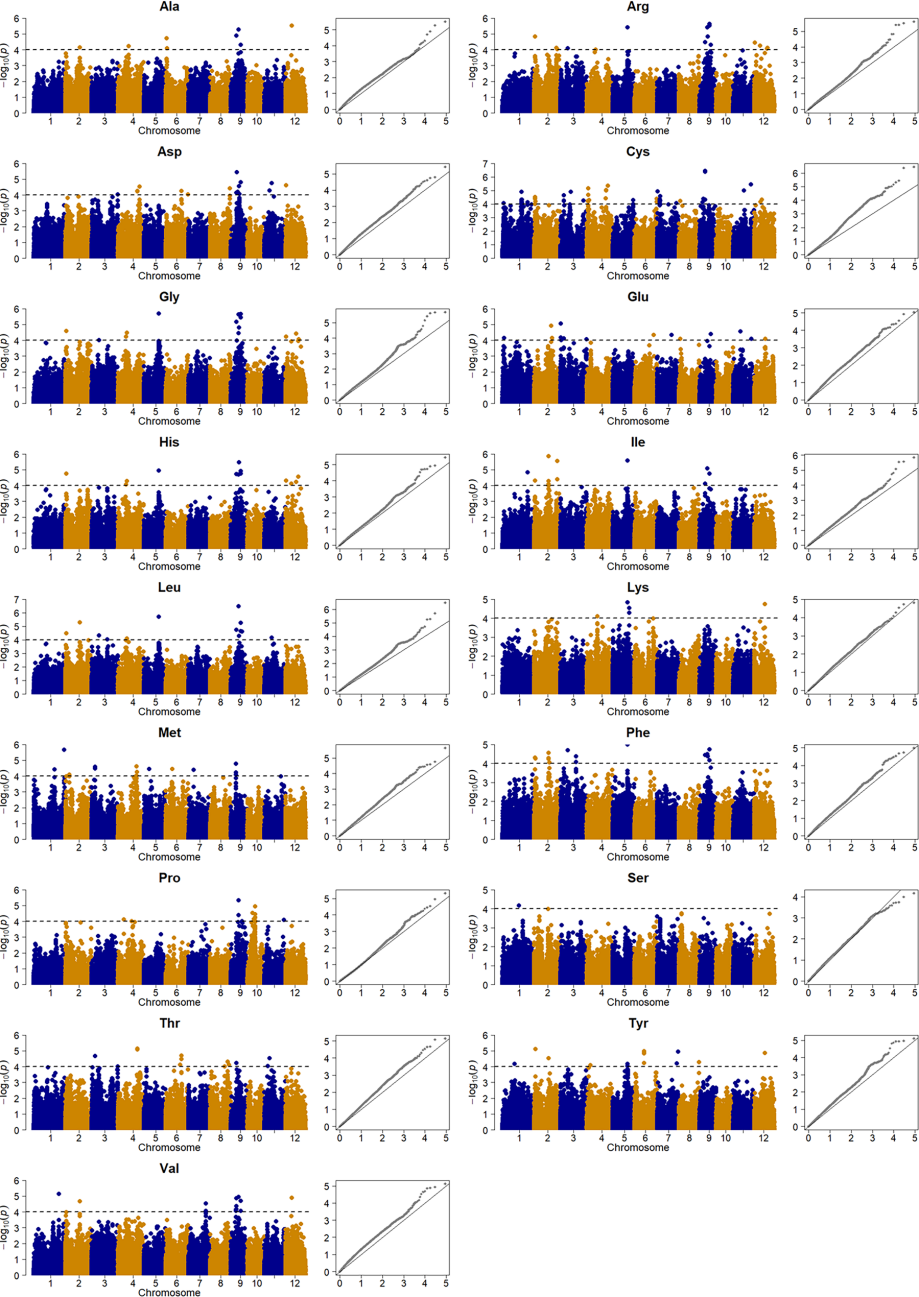
The Manhattan and QQ plots from the genome-wide haplotype association study for the 17 components of amino acid contents in rice

A GWHA study was performed for the 17 AAs to identify genes associated with AAC. The Manhattan and QQ plots suggested that the false positives were controlled properly (Fig. 3). In total, 261 gene-trait associations (GTAs) were identified with a range of 1 GTA for Ser to 67 GTAs for Cys. These GTAs contained 174 unique genes located on all 12 chromosomes with a range of seven genes on chromosomes 8 and 10 to 61 genes on chromosome 9. (Fig. 3, Supplementary Table S3).

Among the 174 genes, 34 genes were associated with at least two components (Supplementary Table S4). Notably, *LOC_Os09g13650* was associated with 11 components of AAC, and *LOC_Os05g34740*, *LOC_Os09g17830* and *LOC_Os09g23334* were associated with eight components of AAC. *LOC_Os09g23380* affected the content of seven AAs including Arg, Asp, Gly, His, Leu, Phe, and Val. *LOC_Os02g03690* and *LOC_Os09g18240* were associated with six components of AAC. *LOC_Os02g33450* affected the content of five AAs including Ala, Ile, Leu, Phe, and Val. Four genes (*LOC_Os03g18810*, *LOC_Os04g22260*, *LOC_Os12g03010* and *LOC_Os12g25140*) were detected to influence the content of four AAs. *LOC_Os09g25650* and *LOC_Os11g17990* were associated with three components of AAC, and the other 20 genes affected the contents of two AAs.

**Fig. 4:**
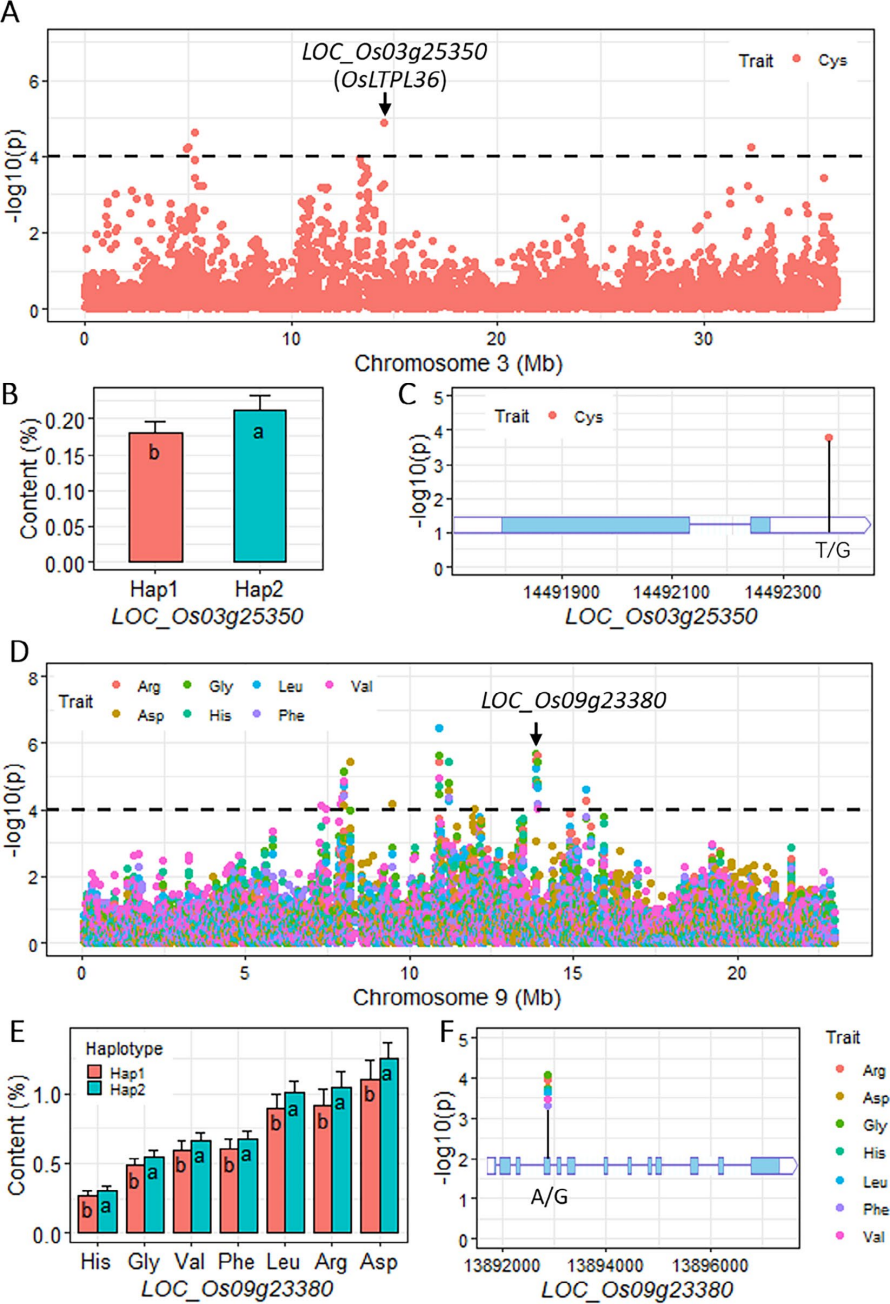
The gene-trait associations detected by the genome-wide haplotype association study and the key natural sequence variants in two candidate genes on chromosomes 3 and 9 of rice. (**A**) The local Manhattan plot on chromosome 3 of genome-wide haplotype association study for Cys. (**B**) The haplotype test of *LOC_Os03g25350* associated with Cys. (**C**) The natural variation of *LOC_Os03g25350* and its association with Cys. (**D**) The local Manhattan plot on chromosome 9 of genome-wide haplotype association study for the seven amino acids. (**E**) The haplotype test of *LOC_Os09g23380* associated with the seven amino acids. (**F**) The natural variation of *LOC_Os09g23380* and its associations with the seven amino acids

We also performed a GWAS using the SNPs within these genes, which allowed us to identify the key natural genetic variations. For example, *OsLTPL36* (*LOC_Os03g25350*), which encodes a lipid transfer protein and has been cloned, affects seed protein content (Fig. 4A). In our study, haplotype analysis showed that *OsLTPL36* is associated with Cys, and the haplotype Hap2 had higher Cys content than Hap1 (Fig. 4B). Moreover, a significant association with SNP S3_14492383 was also detected by GWAS; this SNP is a T/G mutation located in the 3’UTR region of *OsLTPL36* (Fig. 4C). Another case is *LOC_Os09g23380*, a gene that encodes a metallo-beta-lactamase that was found to be associated with seven components of AAC including Arg, Asp, Gly, His, Leu, Phe, and Val (Fig. 4D). The AAC of haplotype Hap2 was higher than that of Hap1 for all seven AAs (Fig. 4E). Within this gene, we detected a significant SNP (S9_13892879), which is a G/A mutation causing a missense variant from Arg to Lys (Fig. 4F).

## Discussion

### Trait Performance

Two major groups of *O. sativa* have historically been recognized for over 2,000 years, and these groups are known as *Xian* and *Geng* (Wang et al. 2018). Student’s *t*-tests showed that there were no significant differences in AAC between the *Xian* and *Geng* subpopulations except Cys, which indicated that the same genetic mechanisms underlie AAC in the two subpopulations. The levels of the different AAs varied substantially, in particular, the levels of the three EAAs - Lys, Met, and Thr, were quite low, and far below the levels for required optimum growth in people. These AA levels are considered to limit the nutritional quality of rice, which should be improved in high quality rice breeding programs (Galili et al. 2016).

### Advantages and Limitations of GWHA Studies

Cloning QTLs that affect complex traits has been a major challenge, because the classical strategy using map-based cloning for QTL cloning is extremely labor intensive and time consuming. In the current study, the haplotypes of genome-wide annotated genes were used to conduct an association study, which allowed us to directly identify candidate genes, and further gene functional validation can be applied using transgenic or gene editing approaches which will save time and effort in QTL cloning. The associations between SNPs within the genes and target traits were also analyzed using traditional GWAS to identify the key natural variations in the genes, and these variations could be further converted to high throughput KASP (kompetitive allele-specific PCR) markers for marker assisted selection in breeding. SNP mutations in gene coding regions often cause changes in gene function that can lead to phenotypic variation; therefore, using SNPs within genes to construct haplotypes to detect candidate genes associated with investigated traits is quite feasible. Therefore, our approach is also applicable for studying other traits to identify candidate genes and functional markers associated with the target traits. However, the SNPs in the downstream and upstream regions of genes were excluded in the current study, and these are also important factors that can affect gene function. As a result, some key natural variations in phenotypic diversity might have been missed. Another limitation was that the SNP calling and gene models were based on the ‘Nipponbare’ reference genome, and the genes that are missing in ‘Nipponbare’ cannot be identified. Thus, more high-quality rice reference genomes will be helpful to solve this problem in future studies.

### Candidate Genes for AAC Detected by GWHA Analysis

In the present study, a total of 174 genes for the 17 components of AAC were detected in a GWHA study. Twelve of these genes that affect rice yield-related agronomic traits or resistance have been cloned; *OsHSP17.0*, *OsRACK1A*, *OsEBF2*, *OsPRX2*, *OsLTPL36*, *OsABCC7*, *MFAP1*, *FON1*, *OsGASR9*, *Hd18*, *OsRRM*, and *OsEMF2b* (Supplementary Table S3). The roles of these genes in AAC have not been reported previously. However, one of these genes, *OsLTPL36*, which encodes a lipid transfer protein, was reported to affect seed protein content. Knocking down the expression of *OsLTPL36* resulted in reduced seed protein content (Wang et al. 2015). In our study, we also detected another four lipid transfer protein genes; *LOC_Os01g59870* and *LOC_Os07g07790* were associated with Cys, and *LOC_Os10g20830* and *LOC_Os10g20890* were associated with Pro (Supplementary Table S3).

Aminotransferases are enzymes that catalyze the amino transamination from an amino donor compound to the carbonyl position of an amino acceptor compound, and they play essential roles in AA metabolism in plants. At present, 13 genes encoding aminotransferases have been cloned in rice; these genes are involved in AA metabolism and also affect rice grain quality, grain size, grain yield, seed set, biotic stress, and abiotic stress (https://ricedata.cn/gene/). In our study, the aminotransferase gene *LOC_Os03g18810* was identified through its association with Phe, Leu, Arg, and Gly (Supplementary Table S4).

### Implications for Rice Breeding

Nutritional quality in rice is a major consideration for consumers. Increasing AAC can enhance rice grain nutritional quality which is beneficial for human health. Nutritional quality in rice is a major consideration for consumers. Increasing AAC can enhance rice grain nutritional quality which is beneficial for human health. To enhance the nutritional quality of rice, the contents of many AAs could be simultaneously improved by identification and characterization of novel genes for AAC. For example, Nguyen et al. (2012) generated five transgenic rice plants with elevated levels of methionine (1.4-fold) and cysteine (2.4-fold) by expressing an *Escherichia coli* serine acetyltransferase isoform gene driven by an ubiquitin promoter. Notably, the transgenic plants also exhibited higher Met, Iso, Leu, and Val contents. In this study, we detected significant positive correlations among almost all the components of AAC, and this was verified by the identification of 34 genes associated with at least two components of AAC in a GWHA analysis. Thus the 34 genes that govern at least two AACs could be further utilized in simultaneously increasing the contents of multiple AAs in rice nutritional quality improvement.

In the current study, we identified nine accessions with the highest grain AAC for 3–14 AAs, and the total AAC was above 12.28% (Table 1); thus, these accessions can be used as parents in rice breeding programs to improve rice nutritional quality. However, high AAC also means high protein content, and too high protein content is associated with reduced palatability and eating quality. One of the solutions is to increase the contents of certain EAAs while maintaining a low protein content. For example, Lys is one the EAAs that beneficial for human health. The content of Lys ranged from 0.35% to 0.64% in the rice association panel. There were 41 accessions in which the Lys content was > 0.54%, and that was the upper limit of grain lysine content in rice reported by Huang et al. (2023). Among the 41 accessions, 32 were from the *Xian* subpopulation and nine were from the *Geng* subpopulation. In particular, the accession IRIS_313.9771 had the highest Lys content (0.64%) but a low total AAC of 9.65%, and is a valuable germplasm resource for breeding high-Lys rice to improve nutritional quality and also maintain eating quality. The accessions with high AAC identified in this study are released cultivars or advanced breeding lines and thus can be directly used as parents in rice breeding programs.

Compared with traditional breeding programs, the combination of conventional breeding and molecular techniques, such as marker-assisted selection (MAS), is a more efficient approach for improving rice grain nutritional quality (Chen et al. 2018). Using a GWHA study enabled us to identify the best haplotypes, which in turn provides genetic resources for rice transgenic breeding or gene-editing breeding. The key natural genetic variations identified here can be further converted to high-throughput KASP markers to use in rice MAS breeding.

## Conclusion

In this study, a GWHA study was conducted for 17 components of AAC in rice using a diverse panel of 164 accessions genotyped with 32 M SNPs derived from the 3 K RGP. The haplotypes of 44,248 genes were constructed using 1,123,603 SNPs, and each gene had 2–38 haplotypes. Through the GWHA study, we detected 261 GTAs involving 174 genes for all of the investigated traits. Among them, twelve genes have been cloned, and 34 genes affected at least two components, indicating that the GWHA study is an efficient approach to identify candidate genes for quantitative traits. These candidate genes, the best haplotypes, and key natural variations affecting AAC provide valuable information for future functional studies and MAS-based breeding to improve rice nutritional quality.

### Electronic Supplementary Material

Below is the link to the electronic supplementary material.

Additional file 1: **Table S1** The origins and group assignments of the 164 rice accessions used in this study. **Table S2** The distribution of the 1,123,603 high-quality SNPs on the 12 rice chromosomes used in the genome-wide haplotype association study. **Table S3** The 261 gene-trait associations detected by genome-wide haplotype association study and their key natural variations. **Table S4** The genes associated with multipe amino acids detected by genome-wide haplotype association study

Additional file 2: Figure S1 A box plot of the 17 components of AAC in the *Xian* and *Geng* rice subpopulations. * indicates that the difference in AAC between *Xian* and *Geng* was significant at the 0.05 level

## Data Availability

The datasets presented in this study can be found in online repositories. The names of the repository/repositories and accession number(s) can be found in the article/Supplementary Material.

## Abbreviations

AAC: amino acid content
AA: amino acid
EAAs: essential amino acids
DAAs: dispensable amino acids
QTL: quantitative trait loci
M-QTLs: main effect QTLs
GWAS: genome-wide association study
RILs: recombinant inbred lines
AK: aspartate kinase
DHPS: dihydrodipicolinate synthase
OsLKR/SDH: lysine-ketoglutarate reductase/saccharopine dehydrogenase
3K RGP: 3,000 Rice Genomes Project
MAF: minor allele frequency
GWHA: genome-wide haplotype association
GTAs: gene-trait associations
PVE: phenotypic variation explained
KASP: kompetitive allele-specific PCR
MAS: marker-assisted selection
