# Supplementary figures and images for: Identification of Candidate Genes that Affect the Contents of 17 Amino Acids in the Rice Grain Using a Genome-Wide Haplotype Association Study
Source: Rice (N Y). 2023 Sep 15;16:40. doi: 10.1186/s12284-023-00658-9 (PMC10504229; doi:10.1186/s12284-023-00658-9)

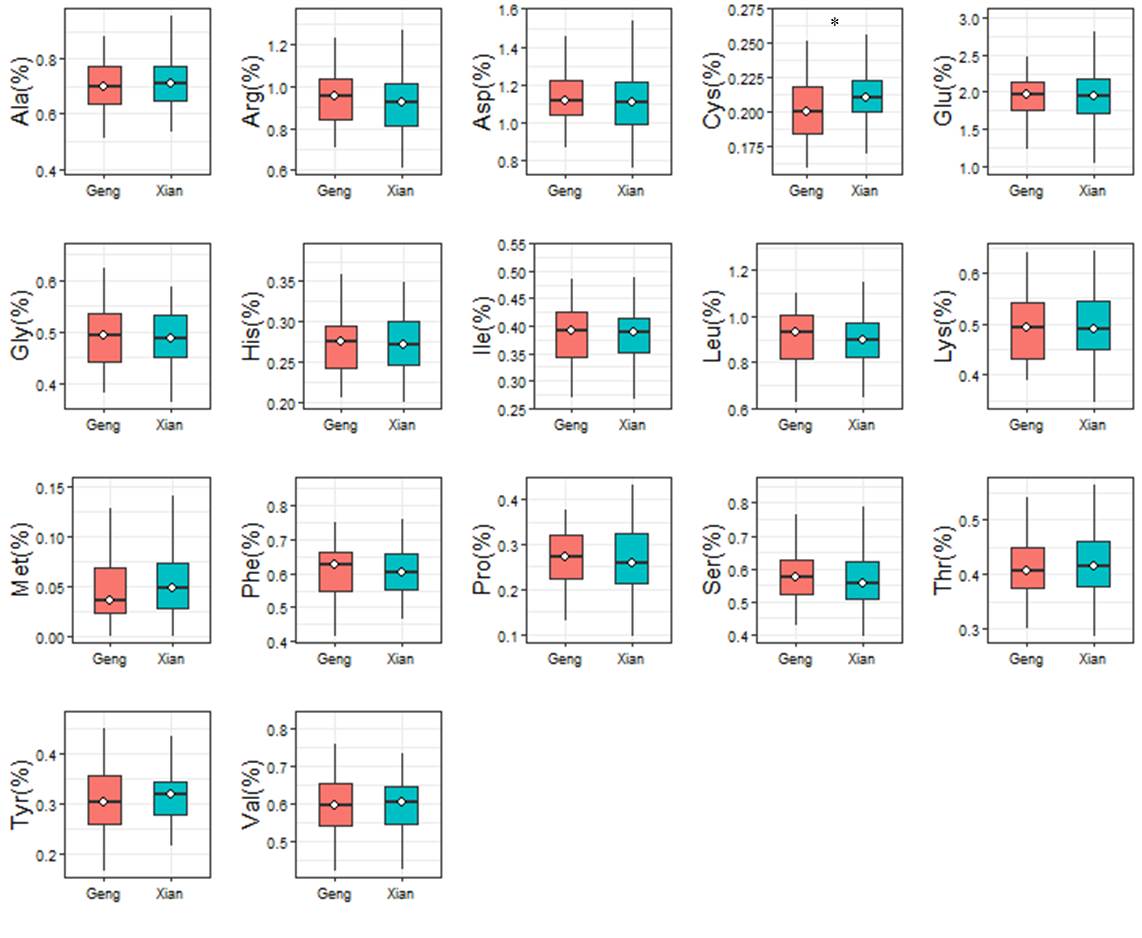

Supplement: Supplementary file 2 — Additional file 2: Figure S1 A box plot of the 17 components of AAC in the Xian and Geng rice subpopulations. * indicates that the difference in AAC between Xian and Geng was significant at the 0.05 level [file 12284_2023_658_MOESM2_ESM.jpg]
